# Distinct coordination geometries in bis­[*N*,*N*-bis­(2-meth­oxy­eth­yl)di­thio­carbamato-κ^2^ *S*,*S*′]di­phenyltin(IV) and bis­[*N*-(2-meth­oxy­eth­yl)-*N*-methyl­dithio­carbamato-κ^2^ *S*,*S*′]di­phenyl­tin(IV): crystal structures and Hirshfeld surface analysis

**DOI:** 10.1107/S2056989016011385

**Published:** 2016-07-19

**Authors:** Rapidah Mohamad, Normah Awang, Mukesh M. Jotani, Edward R. T. Tiekink

**Affiliations:** a Biomedical Science Programme, School of Diagnostic and Applied Health Sciences, Faculty of Health Sciences, Universiti Kebangsaan Malaysia, Jalan Raja Muda Abdul Aziz, 50300 Kuala Lumpur, Malaysia; b Environmental Health and Industrial Safety Programme, School of Diagnostic and Applied Health Sciences, Faculty of Health Sciences, Universiti Kebangsaan Malaysia, Jalan Raja Muda Abdul Aziz, 50300 Kuala Lumpur, Malaysia; c Department of Physics, Bhavan’s Sheth R. A. College of Science, Ahmedabad, Gujarat 380 001, India; d Research Centre for Chemical Crystallography, Faculty of Science and Technology, Sunway University, 47500 Bandar Sunway, Selangor Darul Ehsan, Malaysia

**Keywords:** crystal structure, organotin, di­thio­carbamate, Hirshfeld surface analysis

## Abstract

The crystal and mol­ecular structures of two di­phenyl­tin bis­(di­thio­carbamate)s, [Sn(C_6_H_5_)_2_(C_5_H_10_NOS_2_)_2_], (I), and [Sn(C_6_H_5_)_2_(C_7_H_14_NO_2_S_2_)_2_], (II), are described. In (I), in which the metal atom lies on a twofold rotation axis, the di­thio­carbamate ligand coordinates with approximately equal Sn—S bond lengths and the *ipso*-C atoms of the Sn-bound phenyl groups occupy *cis*-positions in the resulting octa­hedral C_2_S_4_ donor set. A quite distinct coordination geometry is noted in (II), arising as a result of quite disparate Sn—S bond lengths. Here, the four S-donors define a trapezoidal plane with the *ipso*-C atoms lying over the weaker of the Sn—S bonds so that the C_2_S_4_ donor set defines a skewed trapezoidal bipyramid. The packing of (I) features supra­molecular layers in the *ab* plane sustained by methyl­ene-C—H⋯π(Sn–ar­yl) inter­actions; these stack along the *c*-axis direction with no specific inter­actions between them. In (II), supra­molecular chains along the *b*-axis direction are formed by methyl­ene-C—O(ether) inter­actions; these pack with no directional inter­actions between them. A Hirshfeld surface analysis was conducted on both (I) and (II) and revealed the dominance of H⋯H inter­actions contributing to the respective surfaces, *i.e*. >60% in each case, and other features consistent with the description of the mol­ecular packing above.

## Abstract 2

Two distinct coordination geometries, each based on a C_2_S_4_ donor set, are found in the title compounds, being based on an octa­hedron in (C_6_H_5_)_2_Sn(S_2_CN(Me)CH_2_CH_2_OMe)_2_ and a skew trapezoidal bipyramid in (C_6_H_5_)_2_Sn[S_2_CN(CH_2_CH_2_OMe)_2_]_2_.

## Chemical context   

In a review of the applications and structures of tin/organotin di­thio­carbamates (di­thio­carbamate is ^−^S_2_CN*RR*’; *R*, *R*′ = alkyl, ar­yl), two applications were highlighted, namely, their potential biological activity and their utility as single-source precursors for tin sulfide nanoparticles (Tiekink, 2008 ▸). Investigations in both fields continue, *e.g*. as anti-cancer agents (Khan *et al.*, 2014 ▸, 2015 ▸; Kadu *et al.*, 2015 ▸), as anti-microbials (Zia-ur-Rehman *et al.*, 2011 ▸; Ferreira *et al.*, 2012 ▸, 2014 ▸) and as fungicides (Yu *et al.*, 2014 ▸). The use of various tin di­thio­carbamate species as precursors for tin sulfide materials also continues to attract significant attention (Ramasamy *et al.*, 2013 ▸; Lewis *et al.*, 2014 ▸; Kevin *et al.*, 2015 ▸). It was during the course of ongoing studies of the anti-tumour potential of organotin di­thio­carbamates (Khan *et al.*, 2014 ▸, 2015 ▸) that attention was directed towards (2-meth­oxy­eth­yl)di­thio­carbamate derivatives. Herein, the crystal and mol­ecular structures of two di­phenyl­tin derivatives, *viz*. [Sn(C_6_H_5_)_2_(C_5_H_10_NOS_2_)_2_], (I), and [Sn(C_6_H_5_)_2_(C_7_H_14_NOS_2_)_2_], (II), are reported that exhibit quite distinct coordination geometries, along with a Hirshfeld surface analysis to provide more details on the mol­ecular packing.
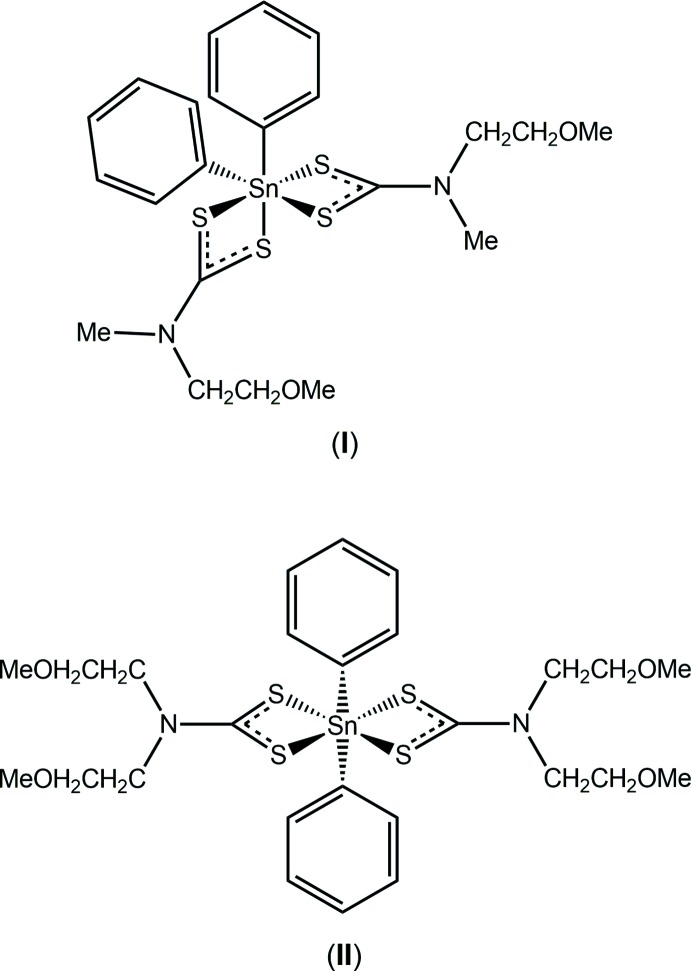


### Structural commentary   

**Figure 1:**
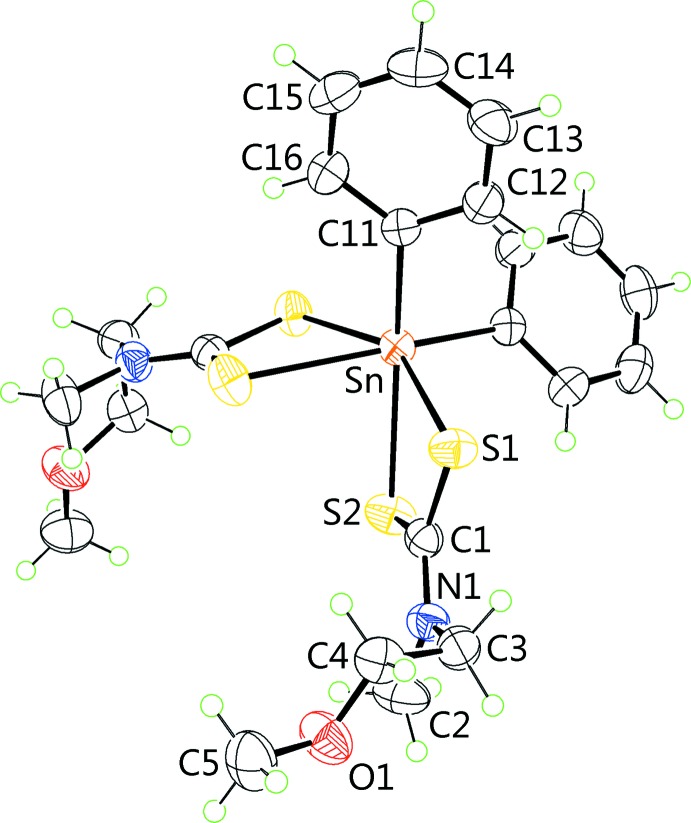
The mol­ecular structure of (I), showing the atom-labelling scheme and displacement ellipsoids at the 50% probability level. Unlabelled atoms are related by the symmetry operation (1 − *x*, *y*, 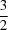 − *z*).

The asymmetric unit of (I) comprises half a mol­ecule as the tin atom is located on a twofold rotation axis, Fig. 1 ▸. The C_2_S_4_ donor set is defined by two chelating di­thio­carbamate ligands and the *ipso*-carbon atoms of the tin-bound phenyl substituents. The difference between the Sn—S_short_ and Sn—S_long_ bond lengths, *i.e*. Δ(Sn—S), is relatively small at 0.06 Å, indicating an essentially symmetric coordination mode. This symmetry is reflected in the near equivalence of the associated C1—S bond lengths with the difference between them being 0.024 Å, Table 1 ▸. The longer Sn—S2 bond is approximately *trans* to the *ipso*-carbon atom. The overall coordination geometry is based on an octa­hedron with the *ipso*-carbon atoms occupying mutually *cis* positions. The meth­oxy­ethyl group is approximately perpendicular to the S_2_CN core as seen in the value of the C1—N1—C3—C4 torsion angle of 93.8 (2)°. The residue itself is almost planar and adopts an extended conformation as seen in the C5—O1—C4—C3 torsion angle of 175.27 (19)°.

**Figure 2:**
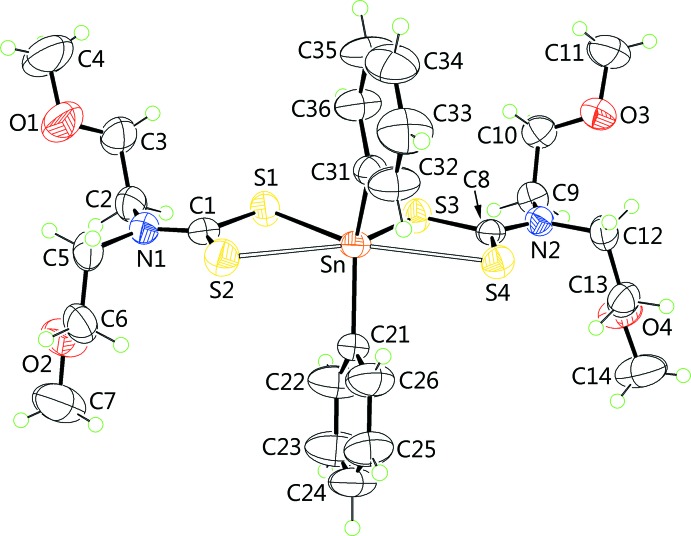
The mol­ecular structure of (II), showing the atom-labelling scheme and displacement ellipsoids at the 50% probability level.

The mol­ecule in (II), Fig. 2 ▸, lies on a general position and has a quite distinct coordination geometry. As for (I), the tin atom is located within a C_2_S_4_ donor set. However, the di­thio­carbamate ligand is coordinating with significantly greater values of ΔS, *i.e*. 0.48 and 0.46 Å for the S1- and S3-ligands, respectively, with the Sn—S_short_ bonds in (II) being shorter than the equivalent bonds in (I) and at the same time, the Sn—S_long_ bonds in (II) being longer than those in (I). An inter­esting consequence of the different modes of coordination of the di­thio­carbamate ligands in the two structures is that the Sn—C_bond_ lengths in (II) are considerably shorter than those in (I), Table 1 ▸. As the di­thio­carbamate anions are approximately co-planar and the more tightly bound S1 and S3 atoms lie to the same side of the mol­ecule, the S_4_ donor atoms define a trapezoidal plane. The tin-bound *ipso*-carbon atoms are disposed over the weaker Sn—S bonds so that the coordination geometry is skewed trapezoidal bipyramidal. Reflecting the significant disparity in the Sn—S bonds, there are large differences in the associated C—S bonds with the shorter of these being associated with the weakly coordinating sulfur atoms, Table 1 ▸. As for (I), the meth­oxy­ethyl groups lie almost perpendicular to the plane through the S_2_CN atoms with the greatest deviation being for the O1-containing residue, *i.e*. the C1—N1—C5—C6 torsion angle is −81.5 (3)°. For each di­thio­carbamate ligand, the residues lie to either side of the S_2_CN plane, and each is as for (I), adopting an almost planar and extended conformation with the O4-residue showing the greatest deviation, albeit marginally, as seen in the C14—O4—C13—C12 torsion angle of 176.3 (2)°.

## Supra­molecular features   

**Figure 3:**
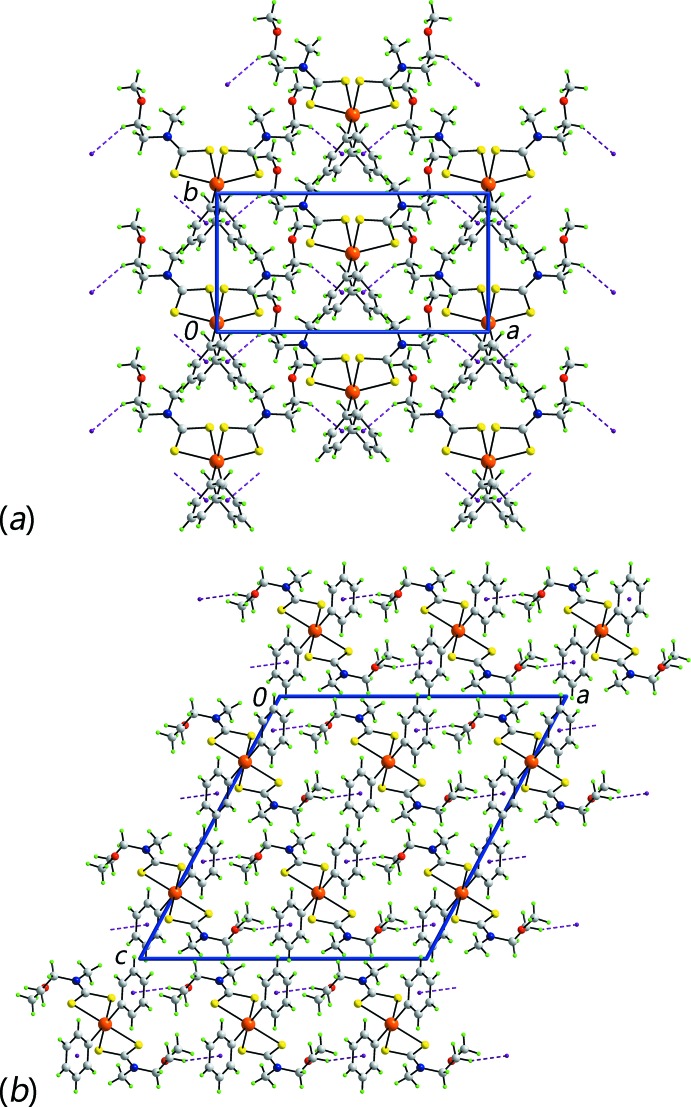
Mol­ecular packing in (I), showing (*a*) a supra­molecular layer in the *ab* plane sustained by methyl­ene-C—H⋯π(Sn-phen­yl) inter­actions (purple dashed lines) and (*b*) a view of the unit-cell contents in projection down the *b* axis.

Geometric parameters characterizing the inter­molecular inter­actions operating in the crystal structures of (I) and (II) are collected in Tables 2 ▸ and 3 ▸, respectively. Based on the distance criteria in *PLATON* (Spek, 2009 ▸), the only significant inter­molecular contact in the mol­ecular packing of (I) is a methyl­ene-C—H⋯π(Sn–ar­yl) inter­action. From symmetry, there are four such inter­actions per mol­ecule so that a two-dimensional supra­molecular layer in the *ab* plane ensues, Fig. 3 ▸ *a*. These stack along the *c* axis being separated by hydro­phobic inter­actions, Fig. 3 ▸ *b*.

**Figure 4:**
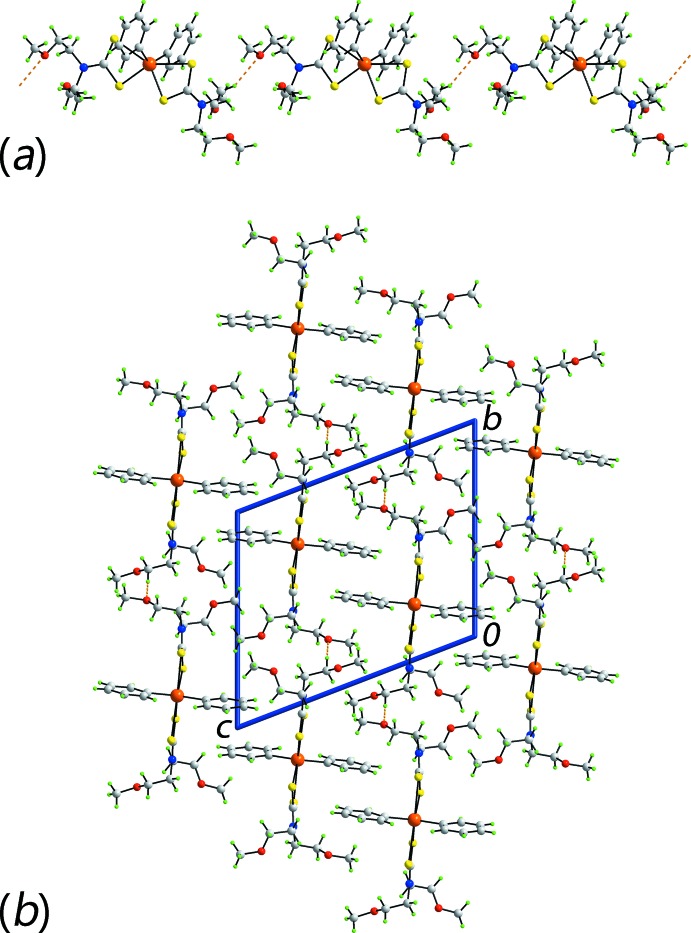
Mol­ecular packing in (II), showing (*a*) a supra­molecular chain along the *b* axis sustained by methyl­ene-C—H⋯O inter­actions (orange dashed lines) and (*b*) a view of the unit-cell contents in projection down the *a* axis.

In the mol­ecular packing of (II), methyl­ene-C—H⋯O inter­actions lead to linear supra­molecular chains along the *b* axis, Fig. 4 ▸ *a*. These pack into the three-dimensional architecture of the crystal with no directional inter­molecular inter­actions between them, Fig. 4 ▸ *b*.

A more detailed analysis of the mol­ecular packing in (I) and (II) is given below in Hirshfeld surface analysis.

## Hirshfeld surface analysis   

Hirshfeld surfaces for (I) and (II) were mapped over *d*_norm_, *d*_e_, shape-index, curvedness and electrostatic potential with the aid of *Crystal Explorer 3.1* (Wolff *et al.*, 2012 ▸). The electrostatic potentials were calculated using *TONTO* (Spackman *et al.*, 2008 ▸; Jayatilaka *et al.*, 2005 ▸), integrated into *Crystal Explorer*, and were mapped on the Hirshfeld surfaces using the STO-3G basis set at Hartree–Fock level of theory over the range ±0.12 au. The contact distances *d*_e_ and *d*_i_ from the Hirshfeld surface to the nearest atom inside and outside, respectively, enables the analysis of the inter­molecular inter­actions through the mapping of *d*_norm_. The combination of *d*_e_ and *d*_i_ in the form of two-dimensional fingerprint plots (McKinnon *et al.*, 2007 ▸) provides a visual summary of inter­molecular contacts in the crystal.

**Figure 5:**
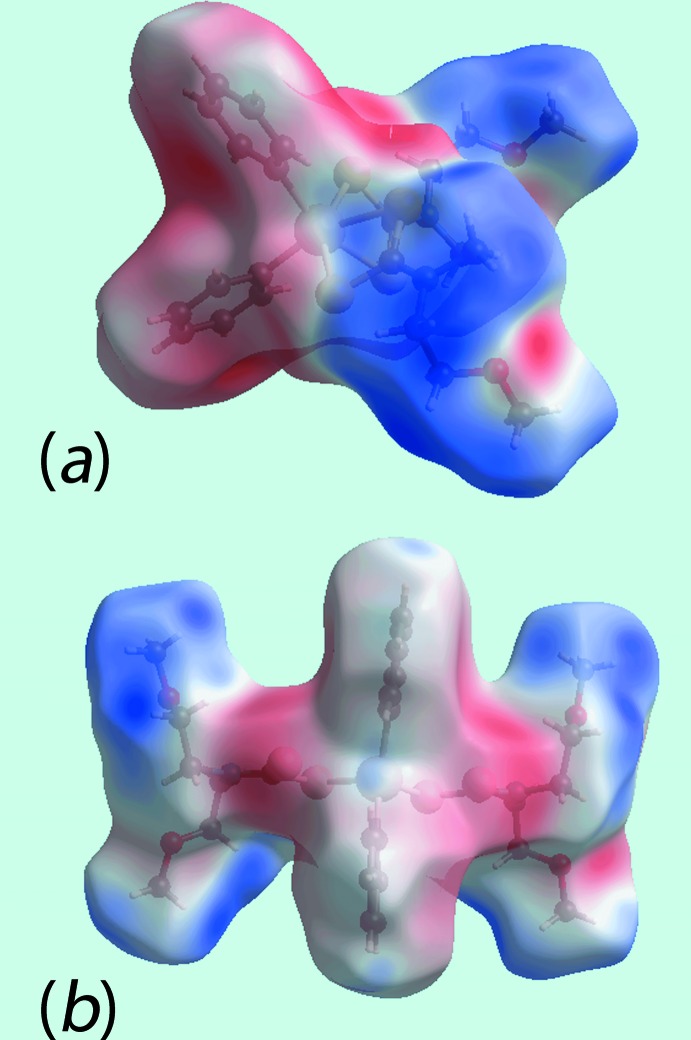
View of Hirshfeld surfaces mapped over the electrostatic potential (the red and blue regions represent negative and positive electrostatic potentials, respectively): (*a*) for (I) and (*b*) for (II).

**Figure 6:**
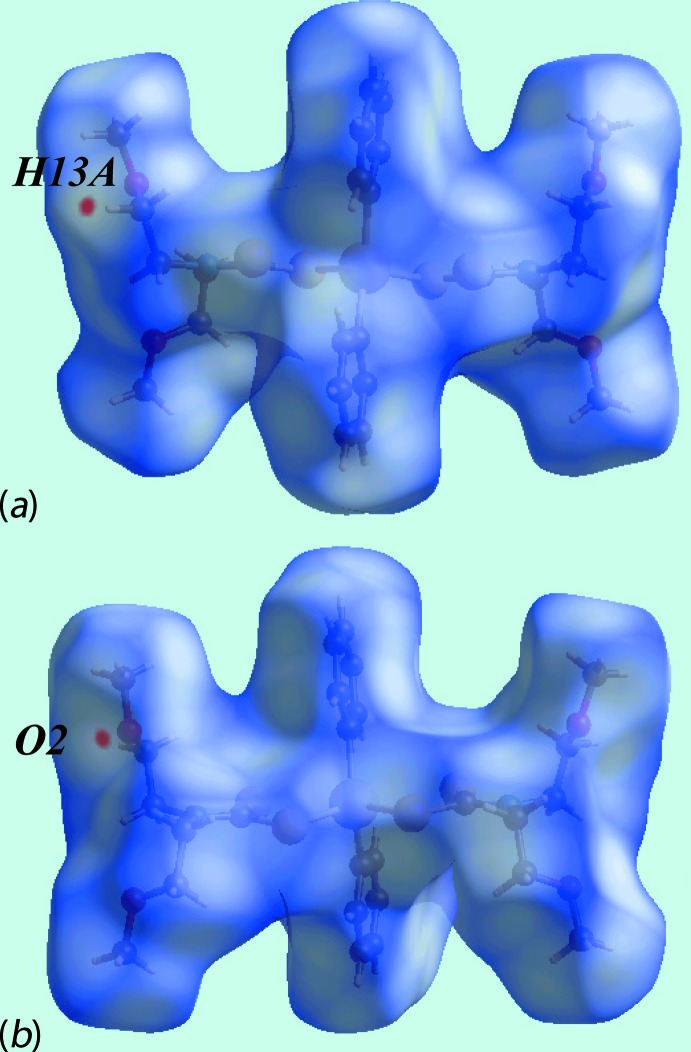
Views of Hirshfeld surfaces mapped over *d*_norm_ for (II).

As evident from Fig. 5 ▸, the Hirshfeld surfaces for (I) and (II) have quite different shapes reflecting the distinctive coordination geometries, and the dark-red and dark-blue regions assigned to negative and positive potentials are localized near the respective functional groups. The absence of conventional hydrogen bonds in the crystal of (I) is consistent with the non-appearance of characteristic red spots in the calculated Hirshfeld surface mapped over *d*_norm_ (not shown). By contrast, in (II), the weak C—H⋯O interaction gives rise to red spots as evident in Fig. 6 ▸.

**Figure 7:**
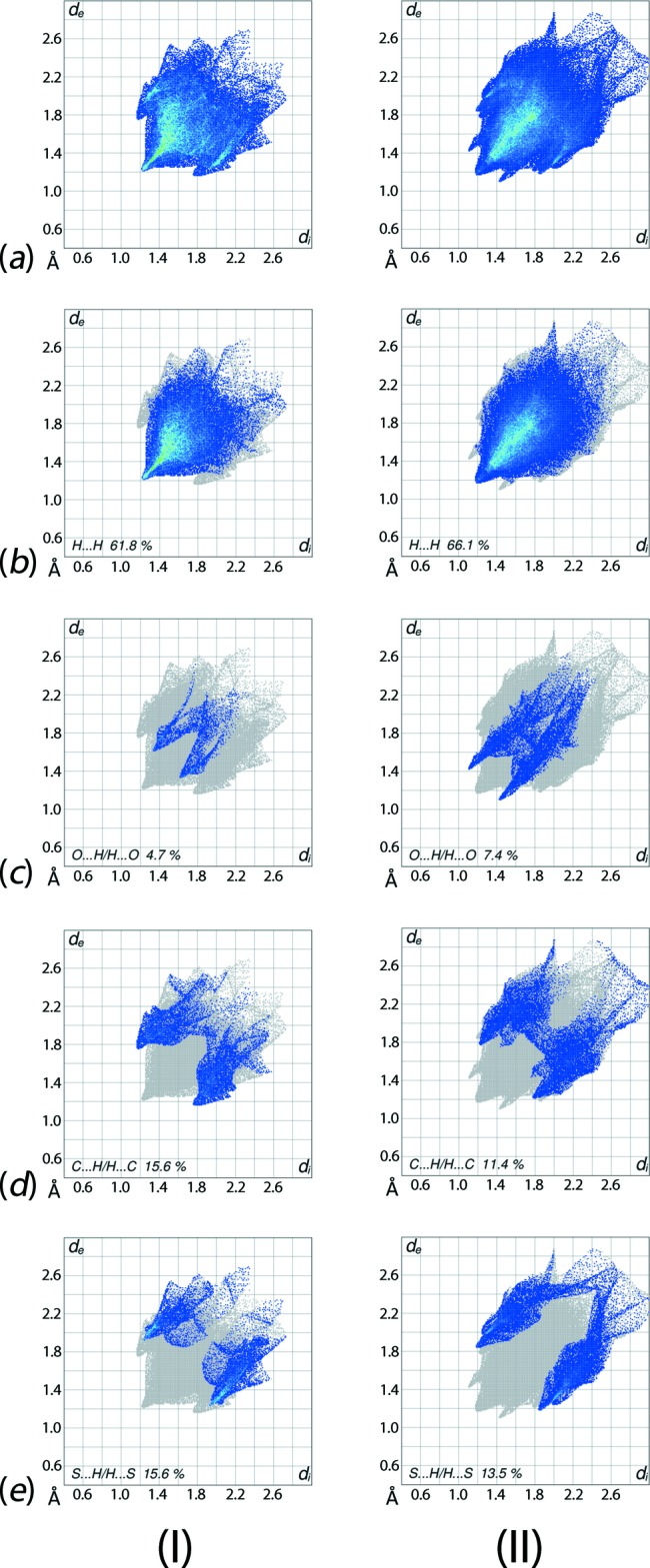
Comparison of the (*a*) complete Hirshfeld surface and full two-dimensional fingerprint plots between (I) and (II), and the plots delineated into (*b*) H⋯H, (*c*) O⋯H/H⋯O, (*d*) C⋯H/H⋯C and (*e*) S⋯H/H⋯S contacts.

The overall two-dimensional fingerprint plots for (I) and (II) and those delineated into H⋯H, O⋯H/H⋯O, C⋯H/H⋯C and S⋯H/H⋯S contacts are illustrated in Fig. 7 ▸; their relative contributions are summarized in Table 4 ▸. The different distribution of points in the overall fingerprint plots for (I) and (II) are due to their different mol­ecular conformations. Also, it is noted that the points are distributed in different (*d*_e_, *d*_i_) ranges, *i.e*. 1.2 to 2.7 Å for (I) and 1.2 to 2.9 Å for (II).

As evident from the data in Table 4 ▸ and the fingerprint plots in Fig. 7 ▸ *b*, H⋯H contacts clearly make the most significant contributions to the Hirshfeld surfaces of both (I) and (II). In the fingerprint plot of (I) delineated into H⋯H contacts (Fig. 7 ▸ *b*), all the points are situated at (*d*_e_, *d*_i_) distances equal to or greater than their van der Waals separations *i.e*. 1.2 Å, reflecting zero propensity to form such inter­molecular contacts. By contrast, for (II), points at (*d*_e_, *d*_i_) distances less than 1.2 Å, with the peak at *d*_e_ = *d*_i_ ∼1.2 Å, resulting from short inter­atomic H⋯H contacts, Table 5 ▸. The 7.4% contribution from O⋯H/H⋯O contacts to the Hirshfeld surface of (II) reflects the presence of an inter­molecular C—H⋯O inter­action and a short inter­atomic O⋯H/H⋯O contact (Table 5 ▸), showing a forceps-like distribution of points with the tips at *d*_e_ + *d*_i_ ∼2.5 Å in Fig. 7 ▸ *c*. The small contribution, *i.e*. 4.7%, due to analogous inter­actions in (I) have a low density of points that are generally masked by other contacts in the plot consistent with a low propensity to form.

**Figure 8:**
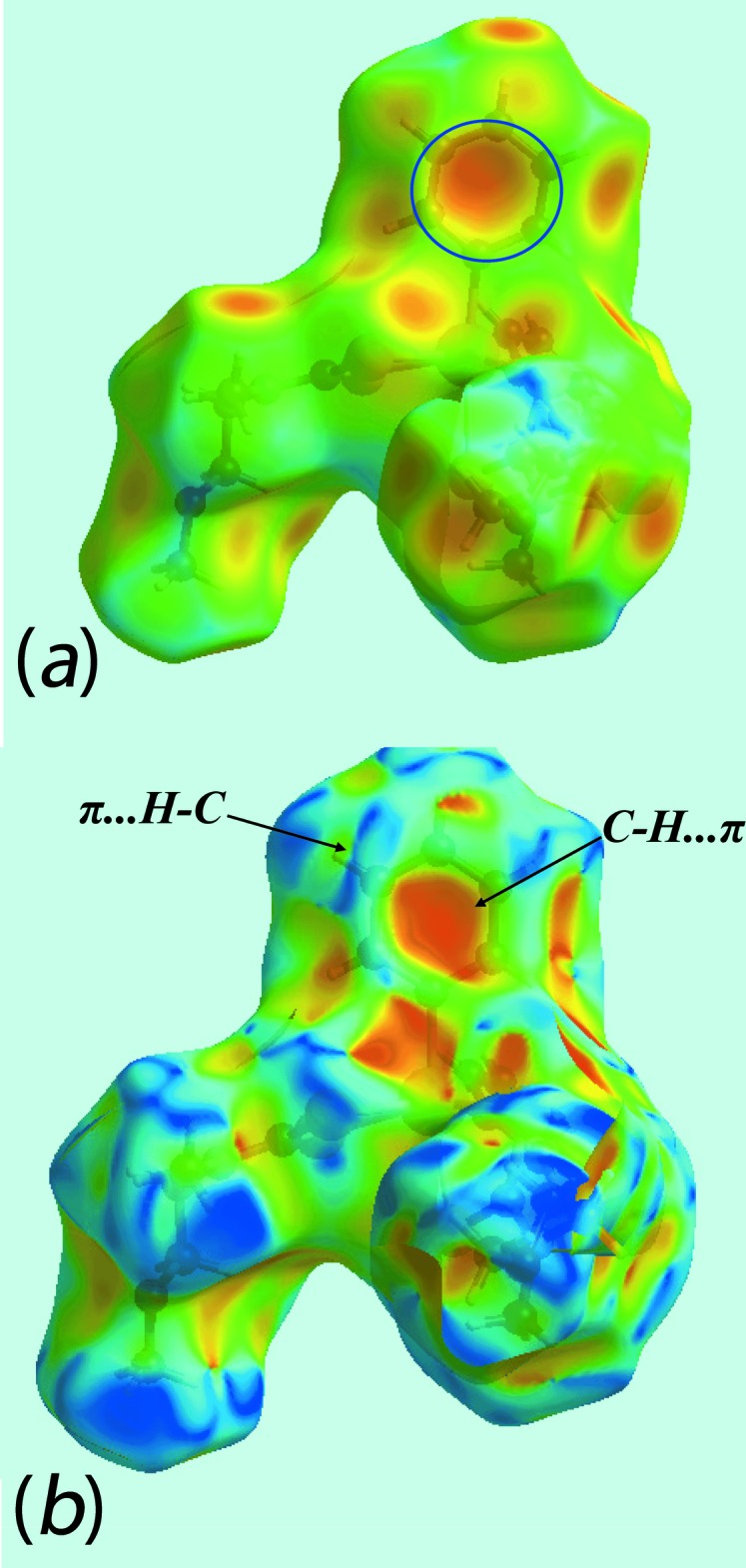
View of the Hirshfeld surfaces for (I), showing (*a*) mapped over *d*_e_ with the pale-orange spot within the blue circle indicating the involvement of the aryl ring in C—H⋯π inter­actions and (*b*) mapped with the shape-index property with the bright-red spot, identified with an arrow, indicating the C—H⋯π inter­action and the blue spots indicating complementary π⋯H—C inter­actions.

The pair of characteristics wings with the edges at *d*_e_ + *d*_i_ ∼2.9 Å in the fingerprint plot delineated into C⋯H/H⋯C contacts for (I) is due to the contribution of methyl­ene-C—H⋯π(Sn–ar­yl) inter­actions, Fig. 7 ▸ *d*. The presence of these inter­actions are also indicated through the pale-orange spots present on the Hirshfeld surface mapped over *d*_e_, shown within the blue circle in Fig. 8 ▸ *a*, and bright-red spots over the front side of shape-indexed surfaces identified with arrows in Fig. 8 ▸ *b*. The reciprocal of these C—H⋯π contacts, *i.e*. π⋯H—C contacts, are seen as blue spots near the ring in Fig. 8 ▸ *b*. The fingerprint plot for (II) delineated into C⋯H/H⋯C contacts has a distinct distribution of points with the (*d*_e_, *d*_i_) distances greater than their van der Waals separations, confirming the absence of these inter­actions, Fig. 7 ▸ *d*. The conformations of di­thio­carbamate ligands in both (I) and (II) limit the sulfur atoms’ ability to form significant S⋯H inter­molecular inter­actions; these atoms are separated at distances greater than their van der Waals radii, *i.e*. 3.0 Å. This observation is despite the nearly symmetrical distributions of points in the respective plots for both (I) and (II), Fig. 7 ▸ *e*, and the significant percentage contributions to their Hirshfeld surfaces (Table 5 ▸).

## Database survey   

**Figure 9:**
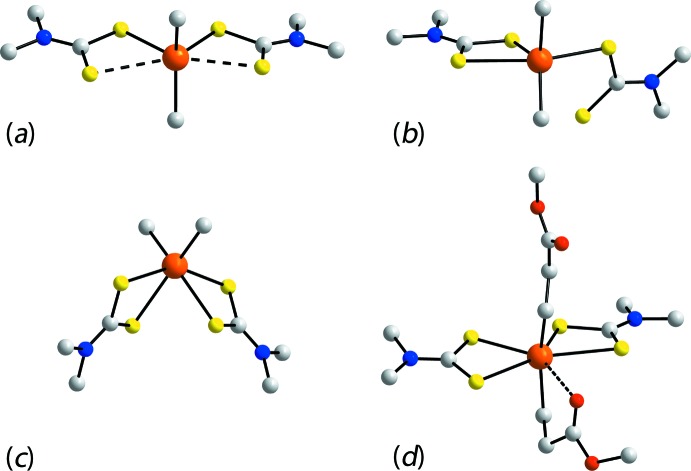
Four structural motifs for mol­ecules of the general composition *R*_2_Sn(S_2_CN*R*′*R*′′)_2_: (*a*) skew trapezoidal bipyramidal, (*b*) five-coordinate trigonal–bipyramidal owing to a monodentate di­thio­carbamate ligand, (*c*) *cis*-octa­hedral and (*d*) seven-coordinate penta­gonal–bipyramidal owing to additional coordination by a heteroatom of the tin-bound residue. In all images, H atoms have been omitted, only the α-C atoms bound to nitro­gen included and, in all but (*d*), only the α-C atom of the tin-bound residues shown.

Given the various applications found for tin di­thio­carbamates, it is not surprising that there exists a relatively large number of structures for this class of compound. Indeed, a search of the Cambridge Structural Database (CSD; Groom *et al.*, 2016 ▸), reveals over 300 ‘hits’. Structural surveys have revealed that very different coordination geometries can arise in the solid state and, even when a common structural motif is adopted, non-systematic variations in geometric parameters are observed (Tiekink, 2008 ▸; Muthalib *et al.*, 2014 ▸). Mononuclear diorganotin bis­(di­thio­carbamate)s, *i.e*. directly related to (I) and (II) described herein, are well represented, there being about 90 examples. Four distinct structural motifs have been noted previously (Tiekink, 2008 ▸), and these are illustrated in Fig. 9 ▸. The two most common motifs are skewed trapezoidal bipyramidal as in (II), Fig. 9 ▸ *a*, and distorted octa­hedral, as in (I), Fig. 9 ▸ *c*. Less common are five-coordinate, trigonal–bipyramidal species, arising as one di­thio­carbamate ligand is monodentate, Fig. 9 ▸ *b*, are found, for example, in the structure of (*t*-Bu)_2_Sn(S_2_CNMe_2_)_2_ (Kim *et al.*, 1987 ▸) and correlated with bulky tin-bound groups, and seven-coordinate species, penta­gonal–bipyramidal, owing to additional coordination by a heteroatom of the tin-bound residue, Fig. 9 ▸ *d*, as for example in the structure of [MeOC(=O)CH_2_CH_2_]_2_Sn(S_2_CNMe)_2_ (Ng *et al.*, 1989 ▸).

There are 16 di­phenyl­tin bis­(di­thio­carbamate) structures included in the CSD and eight of these adopt the motif shown in Fig. 9 ▸ *c*, including both the monoclinic (Lindley & Carr, 1974 ▸) and twofold symmetric tetra­gonal (Hook *et al.*, 1994 ▸) polymorphs of the archetype compound Ph_2_Sn(S_2_CNEt_2_)_2_, and eight adopt the motif shown in Fig. 9 ▸ *a*, including both independent mol­ecules of Ph_2_Sn[S_2_CN(Me)Hex]_2_ (Ramasamy *et al.*, 2013 ▸); the remaining structures are single phase and have one independent mol­ecule. Such an even split suggests a fine energy balance between the adoption of one geometry over the other.

## Synthesis and crystallization   

Synthesis of (I). (2-Meth­oxy­eth­yl)methyl­amine (2 mmol) dissolved in ethanol (10 ml) was stirred in an ice-bath for 30 min. A 25% ammonia solution (1–2 ml) was added to generate a basic solution. Then, a cold ethano­lic solution of carbon di­sulfide (2 mmol) was added to the solution and stirred for about 2 h. Next, di­phenyl­tin(IV) dichloride (1 mmol) dissolved in ethanol was added into the solution and further stirred for 2 h. The precipitate that formed was filtered off and washed a few times with cold ethanol to remove impurities. Finally, the precipitate was dried in a desiccator. Recrystallization was by dissolving the compound with chloro­form and ethanol (2:1 *v*/*v*) ratio. This mixture was allowed to slowly evaporate at room temperature yielding colourless crystals of (I). m.p. 382–384 K. Yield: 78%. IR (cm^−1^): 1,497 ν(C—N), 988 ν(C—S), 523 ν(Sn—C), 389 ν(Sn—S). ^1^H NMR (CDCl_3_): δ 7.28–8.00 (5H, Sn–Ph), 3.97 (2H, OCH_2_), 3.69 (2H, NCH_2_), 3.44 (3H, NMe), 3.36 (3H, OCH_3_). ^13^C NMR (CDCl_3_): δ 199.88 (S_2_C), 128.24–151.24 (Sn–Ar), 69.96 (OCH_2_), 59.06 (NCH_2_), 57.84 (OCH_3_), 45.45 (NCH_3_).

Compound (II) was prepared and recrystallized in essentially the same manner but using bis­(2-meth­oxy­eth­yl)amine (10 mmol) in place of (2-meth­oxy­eth­yl)methyl­amine. m.p. 333–335 K. Yield: 76%. IR (cm^−1^): 1,482 ν(C—N), 985 ν(C—S), 571 ν(Sn—C), 381 ν(Sn—S). ^1^H NMR (CDCl_3_): 7.38–7.89 (5H, Sn–Ph), 4.07 (2H, OCH_2_), 3.77 (2H, NCH_2_), 3.35 (OCH_3_). ^13^C NMR (CDCl_3_): δ 200.16 (S_2_C), 128.26–150.89 (Sn–Ar), 69.90 (OCH_2_), 59.02 (NCH_2_), 56.72 (OCH_3_).

## Refinement   

Crystal data, data collection and structure refinement details are summarized in Table 6 ▸. Carbon-bound H-atoms were placed in calculated positions (C—H = 0.93–0.97 Å) and were included in the refinement in the riding model approximation, with *U*_iso_(H) set to 1.2–1.5*U*_eq_(C).

## Supplementary Material

Crystal structure: contains datablock(s) I, II, global. DOI: 10.1107/S2056989016011385/hb7599sup1.cif

Structure factors: contains datablock(s) I. DOI: 10.1107/S2056989016011385/hb7599Isup2.hkl

Structure factors: contains datablock(s) II. DOI: 10.1107/S2056989016011385/hb7599IIsup3.hkl

CCDC references: 1492701, 1492700

Additional supporting information:  crystallographic information; 3D view; checkCIF report

## Figures and Tables

**Table 1:** Geometric data (Å, °) for (I) and (II)

Parameter	(I)	(II)
Sn—S1	2.6071 (6)	2.5060 (6)
Sn—S2	2.6653 (6)	2.9875 (6)
Sn—S3	–	2.5230 (6)
Sn—S4	–	2.9800 (6)
Sn—C11	2.1677 (18)	–
Sn—C21	–	2.131 (2)
Sn—C31	–	2.124 (2)
C1—S1	1.7311 (19)	1.756 (2)
C1—S2	1.7067 (19)	1.692 (2)
C8—S3	–	1.752 (2)
C8—S4	–	1.692 (2)
S1^i^—Sn—S2^i^	67.742 (17)	64.922 (18)
S3—Sn—S4	–	64.591 (16)
S1—Sn—S1^i^	152.00 (2)	–
S2^i^—Sn—C11^i^	159.03 (5)	–
S1—Sn—S3	–	82.873 (18)
S2—Sn—S4	–	147.642 (17)
C—Sn—C	100.07 (10)	130.12 (9)

Symmetry code: (i) 1 − *x*, *y*, 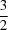 − *z*.

**Table 2:** Hydrogen-bond geometry (Å, °) for (I) *Cg*1 is the centroid of the C11–C16 phenyl ring.

*D*—H⋯*A*	*D*—H	H⋯*A*	*D*⋯*A*	*D*—H⋯*A*
C4—H4*A*⋯*Cg*1^i^	0.97	2.86	3.730 (3)	150

Symmetry code: (i) 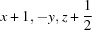.

**Table 3:** Hydrogen-bond geometry (Å, °) for (II)

*D*—H⋯*A*	*D*—H	H⋯*A*	*D*⋯*A*	*D*—H⋯*A*
C13—H13*A*⋯O2^i^	0.97	2.52	3.404 (4)	151

Symmetry code: (i) 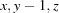.

**Table 4:** Percentage contribution of the different inter­molecular contacts to the Hirshfeld surface in (I) and (II)

Contact	% contribution in (I)	% contribution in (II)
H⋯H	61.8	66.1
C⋯H/H⋯C	15.6	11.4
O⋯H/H⋯O	4.7	7.4
S⋯H/H⋯S	15.6	13.5
C⋯S/S⋯C	1.3	0.0
N⋯H/H⋯N	1.0	0.4
C⋯C	0.0	1.0
S⋯S	0.0	0.1
C⋯O/O⋯C	0.0	0.1

**Table 5:** Short inter­atomic contacts in (II)

Contact	distance	symmetry operation
O4⋯H6*B*	2.69	−1 − *x*, *y*, *z*
H7*C*⋯H14*B*	2.37	1 + *x*, *y*, *z*
H10*B*⋯H34	2.36	1 − *x*, −*y*, −*z*

**Table 6:** Experimental details

	(I)	(II)
Crystal data		
Chemical formula	[Sn(C_6_H_5_)_2_(C_5_H_10_NOS_2_)_2_]	[Sn(C_6_H_5_)_2_(C_7_H_14_NO_2_S_2_)_2_]
*M*_r_	601.41	689.51
Crystal system, space group	Monoclinic, *C*2/*c*	Triclinic, *P* 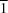
Temperature (K)	293	293
*a*, *b*, *c* (Å)	18.3808 (14), 8.2809 (4), 19.083 (3)	7.4386 (4), 14.3334 (8), 16.5398 (10)
α, β, γ (°)	90, 118.071 (8), 90	110.320 (5), 91.282 (5), 101.865 (4)
*V* (Å^3^)	2562.9 (5)	1609.93 (17)
*Z*	4	2
Radiation type	Mo *K*α	Mo *K*α
μ (mm^−1^)	1.34	1.09
Crystal size (mm)	0.25 × 0.25 × 0.20	0.30 × 0.25 × 0.25

Data collection		
Diffractometer	Agilent Technologies SuperNova Dual diffractometer with Atlas detector	Agilent Technologies SuperNova Dual diffractometer with Atlas detector
Absorption correction	Multi-scan (*CrysAlis PRO*; Agilent, 2015 ▸)	Multi-scan (*CrysAlis PRO*; Agilent, 2015 ▸)
*T*_min_, *T*_max_	0.815, 1.000	0.756, 1.000
No. of measured, independent and observed [*I* > 2σ(*I*)] reflections	7357, 3383, 3051	17063, 8354, 6973
*R*_int_	0.025	0.035
(sin θ/λ)_max_ (Å^−1^)	0.712	0.712

Refinement		
*R*[*F* ^2^ > 2σ(*F* ^2^)], *wR*(*F* ^2^), *S*	0.026, 0.063, 1.07	0.031, 0.078, 1.06
No. of reflections	3383	8354
No. of parameters	143	338
H-atom treatment	H-atom parameters constrained	H-atom parameters constrained
Δρ_max_, Δρ_min_ (e Å^−3^)	0.47, −0.30	0.66, −0.56

Computer programs: *CrysAlis PRO* (Agilent, 2015 ▸), *SHELXL97* (Sheldrick, 2008 ▸), *SHELXL2014* (Sheldrick, 2015 ▸), *ORTEP-3 for Windows* (Farrugia, 2012 ▸), *DIAMOND* (Brandenburg, 2006 ▸) and *publCIF* (Westrip, 2010 ▸).

## supplementary crystallographic information

### (I) Bis[N,N-bis(2-methoxyethyl)dithiocarbamato-κ2S,S']diphenyltin(IV) Crystal data

**Table:**

[Sn(C_6_H_5_)_2_(C_5_H_10_NOS_2_)_2_]	*F*(000) = 1224
*M**_r_* = 601.41	*D*_x_ = 1.559 Mg m^−^^3^
Monoclinic, *C*2/*c*	Mo *K*α radiation, λ = 0.71073 Å
*a* = 18.3808 (14) Å	Cell parameters from 3633 reflections
*b* = 8.2809 (4) Å	θ = 4.2–29.9°
*c* = 19.083 (3) Å	µ = 1.34 mm^−^^1^
β = 118.071 (8)°	*T* = 293 K
*V* = 2562.9 (5) Å^3^	Block, colourless
*Z* = 4	0.25 × 0.25 × 0.20 mm

### (I) Bis[N,N-bis(2-methoxyethyl)dithiocarbamato-κ2S,S']diphenyltin(IV) Data collection

**Table:**

Agilent Technologies SuperNova Dual diffractometer with Atlas detector	3383 independent reflections
Radiation source: SuperNova (Mo) X-ray Source	3051 reflections with *I* > 2σ(*I*)
Mirror monochromator	*R*_int_ = 0.025
Detector resolution: 10.4041 pixels mm^-1^	θ_max_ = 30.4°, θ_min_ = 4.0°
ω scan	*h* = −26→18
Absorption correction: multi-scan (CrysAlis PRO; Agilent, 2015)	*k* = −11→11
*T*_min_ = 0.815, *T*_max_ = 1.000	*l* = −25→26
7357 measured reflections	

### (I) Bis[N,N-bis(2-methoxyethyl)dithiocarbamato-κ2S,S']diphenyltin(IV) Refinement

**Table:**

Refinement on *F*^2^	0 restraints
Least-squares matrix: full	Hydrogen site location: inferred from neighbouring sites
*R*[*F*^2^ > 2σ(*F*^2^)] = 0.026	H-atom parameters constrained
*wR*(*F*^2^) = 0.063	*w* = 1/[σ^2^(*F*_o_^2^) + (0.0284*P*)^2^ + 0.8262*P*] where *P* = (*F*_o_^2^ + 2*F*_c_^2^)/3
*S* = 1.07	(Δ/σ)_max_ = 0.001
3383 reflections	Δρ_max_ = 0.47 e Å^−^^3^
143 parameters	Δρ_min_ = −0.30 e Å^−^^3^

### (I) Bis[N,N-bis(2-methoxyethyl)dithiocarbamato-κ2S,S']diphenyltin(IV) Special details

**Table:**

Geometry. All esds (except the esd in the dihedral angle between two l.s. planes) are estimated using the full covariance matrix. The cell esds are taken into account individually in the estimation of esds in distances, angles and torsion angles; correlations between esds in cell parameters are only used when they are defined by crystal symmetry. An approximate (isotropic) treatment of cell esds is used for estimating esds involving l.s. planes.

### (I) Bis[N,N-bis(2-methoxyethyl)dithiocarbamato-κ2S,S']diphenyltin(IV) Fractional atomic coordinates and isotropic or equivalent isotropic displacement parameters (Å^2^)

**Table:**

	*x*	*y*	*z*	*U*_iso_*/*U*_eq_	
Sn	0.5000	0.56549 (2)	0.7500	0.03252 (7)	
S1	0.65567 (3)	0.64166 (6)	0.82876 (3)	0.04210 (12)	
S2	0.52661 (3)	0.81055 (7)	0.84973 (4)	0.04728 (13)	
O1	0.77790 (11)	1.16594 (19)	0.88988 (10)	0.0563 (4)	
N1	0.68188 (11)	0.90857 (19)	0.91561 (10)	0.0391 (4)	
C1	0.62737 (11)	0.8001 (2)	0.87001 (11)	0.0350 (4)	
C2	0.65719 (17)	1.0454 (3)	0.94849 (16)	0.0568 (6)	
H2A	0.6227	1.1170	0.9063	0.068*	
H2B	0.7054	1.1024	0.9858	0.068*	
H2C	0.6273	1.0063	0.9749	0.068*	
C3	0.76876 (13)	0.9020 (3)	0.93400 (13)	0.0458 (5)	
H3A	0.7836	0.7905	0.9316	0.055*	
H3B	0.8025	0.9398	0.9879	0.055*	
C4	0.78779 (13)	1.0011 (3)	0.87883 (13)	0.0464 (5)	
H4A	0.8440	0.9808	0.8893	0.056*	
H4B	0.7511	0.9709	0.8243	0.056*	
C5	0.80091 (16)	1.2685 (3)	0.84467 (16)	0.0630 (7)	
H5A	0.8589	1.2570	0.8620	0.095*	
H5B	0.7890	1.3784	0.8516	0.095*	
H5C	0.7705	1.2398	0.7896	0.095*	
C11	0.52133 (11)	0.3974 (2)	0.67451 (11)	0.0337 (4)	
C12	0.57526 (14)	0.2710 (3)	0.71091 (13)	0.0470 (5)	
H12	0.6002	0.2605	0.7659	0.056*	
C13	0.59275 (15)	0.1597 (3)	0.66667 (15)	0.0552 (6)	
H13	0.6294	0.0758	0.6920	0.066*	
C14	0.55615 (14)	0.1732 (3)	0.58582 (15)	0.0525 (6)	
H14	0.5679	0.0986	0.5561	0.063*	
C15	0.50225 (14)	0.2964 (3)	0.54873 (13)	0.0512 (5)	
H15	0.4771	0.3052	0.4937	0.061*	
C16	0.48494 (13)	0.4085 (3)	0.59275 (12)	0.0425 (4)	
H16	0.4483	0.4923	0.5669	0.051*	

### (I) Bis[N,N-bis(2-methoxyethyl)dithiocarbamato-κ2S,S']diphenyltin(IV) Atomic displacement parameters (Å^2^)

**Table:**

	*U*^11^	*U*^22^	*U*^33^	*U*^12^	*U*^13^	*U*^23^
Sn	0.03241 (10)	0.03268 (10)	0.03421 (10)	0.000	0.01712 (7)	0.000
S1	0.0349 (2)	0.0430 (3)	0.0492 (3)	0.0002 (2)	0.0205 (2)	−0.0098 (2)
S2	0.0413 (3)	0.0423 (3)	0.0667 (4)	−0.0031 (2)	0.0324 (3)	−0.0114 (2)
O1	0.0732 (11)	0.0438 (8)	0.0706 (11)	−0.0030 (8)	0.0492 (9)	0.0028 (7)
N1	0.0423 (9)	0.0359 (8)	0.0404 (9)	−0.0064 (7)	0.0205 (7)	−0.0038 (7)
C1	0.0380 (9)	0.0340 (9)	0.0363 (9)	−0.0004 (8)	0.0202 (8)	0.0018 (7)
C2	0.0753 (17)	0.0458 (12)	0.0653 (15)	−0.0149 (12)	0.0464 (14)	−0.0188 (11)
C3	0.0362 (10)	0.0428 (11)	0.0470 (12)	−0.0037 (9)	0.0102 (8)	0.0021 (9)
C4	0.0399 (10)	0.0475 (11)	0.0539 (12)	−0.0014 (10)	0.0238 (9)	−0.0044 (10)
C5	0.0649 (16)	0.0613 (15)	0.0704 (17)	−0.0060 (13)	0.0381 (13)	0.0137 (13)
C11	0.0331 (9)	0.0349 (9)	0.0345 (9)	−0.0020 (8)	0.0171 (7)	−0.0019 (7)
C12	0.0529 (12)	0.0457 (11)	0.0374 (10)	0.0098 (10)	0.0169 (9)	0.0000 (9)
C13	0.0552 (13)	0.0432 (12)	0.0634 (15)	0.0122 (11)	0.0248 (11)	−0.0053 (10)
C14	0.0552 (13)	0.0515 (13)	0.0627 (14)	−0.0112 (11)	0.0377 (11)	−0.0230 (11)
C15	0.0549 (13)	0.0636 (14)	0.0371 (11)	−0.0127 (12)	0.0234 (9)	−0.0111 (10)
C16	0.0391 (10)	0.0483 (11)	0.0371 (10)	0.0014 (9)	0.0153 (8)	0.0016 (8)

### (I) Bis[N,N-bis(2-methoxyethyl)dithiocarbamato-κ2S,S']diphenyltin(IV) Geometric parameters (Å, º)

**Table:**

Sn—C11	2.1677 (18)	C3—H3B	0.9700
Sn—C11^i^	2.1678 (18)	C4—H4A	0.9700
Sn—S1	2.6071 (6)	C4—H4B	0.9700
Sn—S1^i^	2.6071 (6)	C5—H5A	0.9600
Sn—S2^i^	2.6653 (6)	C5—H5B	0.9600
Sn—S2	2.6653 (6)	C5—H5C	0.9600
S1—C1	1.7311 (19)	C11—C16	1.381 (3)
S2—C1	1.7067 (19)	C11—C12	1.383 (3)
O1—C4	1.406 (3)	C12—C13	1.386 (3)
O1—C5	1.410 (3)	C12—H12	0.9300
N1—C1	1.322 (2)	C13—C14	1.367 (3)
N1—C3	1.466 (3)	C13—H13	0.9300
N1—C2	1.467 (3)	C14—C15	1.365 (3)
C2—H2A	0.9600	C14—H14	0.9300
C2—H2B	0.9600	C15—C16	1.386 (3)
C2—H2C	0.9600	C15—H15	0.9300
C3—C4	1.500 (3)	C16—H16	0.9300
C3—H3A	0.9700		
			
C11—Sn—C11^i^	100.07 (10)	N1—C3—H3B	108.9
C11—Sn—S1	92.63 (5)	C4—C3—H3B	108.9
C11^i^—Sn—S1	105.36 (5)	H3A—C3—H3B	107.7
C11—Sn—S1^i^	105.36 (5)	O1—C4—C3	109.68 (18)
C11^i^—Sn—S1^i^	92.63 (5)	O1—C4—H4A	109.7
S1—Sn—S1^i^	152.00 (2)	C3—C4—H4A	109.7
C11—Sn—S2^i^	92.54 (5)	O1—C4—H4B	109.7
C11^i^—Sn—S2^i^	159.03 (5)	C3—C4—H4B	109.7
S1—Sn—S2^i^	90.591 (19)	H4A—C4—H4B	108.2
S1^i^—Sn—S2^i^	67.742 (17)	O1—C5—H5A	109.5
C11—Sn—S2	159.03 (5)	O1—C5—H5B	109.5
C11^i^—Sn—S2	92.54 (5)	H5A—C5—H5B	109.5
S1—Sn—S2	67.744 (17)	O1—C5—H5C	109.5
S1^i^—Sn—S2	90.590 (19)	H5A—C5—H5C	109.5
S2^i^—Sn—S2	80.82 (3)	H5B—C5—H5C	109.5
C1—S1—Sn	87.84 (6)	C16—C11—C12	118.01 (18)
C1—S2—Sn	86.46 (7)	C16—C11—Sn	124.38 (14)
C4—O1—C5	113.31 (18)	C12—C11—Sn	117.61 (14)
C1—N1—C3	122.35 (17)	C11—C12—C13	120.9 (2)
C1—N1—C2	121.01 (18)	C11—C12—H12	119.5
C3—N1—C2	116.62 (18)	C13—C12—H12	119.5
N1—C1—S2	121.35 (15)	C14—C13—C12	120.1 (2)
N1—C1—S1	121.16 (14)	C14—C13—H13	120.0
S2—C1—S1	117.49 (11)	C12—C13—H13	120.0
N1—C2—H2A	109.5	C15—C14—C13	119.87 (19)
N1—C2—H2B	109.5	C15—C14—H14	120.1
H2A—C2—H2B	109.5	C13—C14—H14	120.1
N1—C2—H2C	109.5	C14—C15—C16	120.3 (2)
H2A—C2—H2C	109.5	C14—C15—H15	119.9
H2B—C2—H2C	109.5	C16—C15—H15	119.9
N1—C3—C4	113.51 (17)	C11—C16—C15	120.8 (2)
N1—C3—H3A	108.9	C11—C16—H16	119.6
C4—C3—H3A	108.9	C15—C16—H16	119.6
			
C3—N1—C1—S2	179.88 (15)	C5—O1—C4—C3	175.27 (19)
C2—N1—C1—S2	−2.0 (3)	N1—C3—C4—O1	67.0 (2)
C3—N1—C1—S1	0.0 (3)	C16—C11—C12—C13	0.5 (3)
C2—N1—C1—S1	178.13 (16)	Sn—C11—C12—C13	−179.24 (17)
Sn—S2—C1—N1	173.67 (16)	C11—C12—C13—C14	−0.4 (4)
Sn—S2—C1—S1	−6.47 (10)	C12—C13—C14—C15	−0.1 (3)
Sn—S1—C1—N1	−173.53 (16)	C13—C14—C15—C16	0.4 (3)
Sn—S1—C1—S2	6.60 (10)	C12—C11—C16—C15	−0.2 (3)
C1—N1—C3—C4	93.8 (2)	Sn—C11—C16—C15	179.59 (16)
C2—N1—C3—C4	−84.4 (2)	C14—C15—C16—C11	−0.3 (3)

Symmetry code: (i) −*x*+1, *y*, −*z*+3/2.

### (I) Bis[N,N-bis(2-methoxyethyl)dithiocarbamato-κ2S,S']diphenyltin(IV) Hydrogen-bond geometry (Å, º)

Cg1 is the centroid of the C11–C16 phenyl ring.

**Table:**

*D*—H···*A*	*D*—H	H···*A*	*D*···*A*	*D*—H···*A*
C4—H4*A*···*Cg*1^ii^	0.97	2.86	3.730 (3)	150

Symmetry code: (ii) *x*+1, −*y*, *z*+1/2.

### (II) Bis[N-(2-methoxyethyl)-N-methyldithiocarbamato-κ2S,S']diphenyltin(IV) Crystal data

**Table:**

[Sn(C_6_H_5_)_2_(C_7_H_14_NO_2_S_2_)_2_]	*Z* = 2
*M**_r_* = 689.51	*F*(000) = 708
Triclinic, *P*1	*D*_x_ = 1.422 Mg m^−^^3^
*a* = 7.4386 (4) Å	Mo *K*α radiation, λ = 0.71073 Å
*b* = 14.3334 (8) Å	Cell parameters from 6877 reflections
*c* = 16.5398 (10) Å	θ = 3.8–29.7°
α = 110.320 (5)°	µ = 1.09 mm^−^^1^
β = 91.282 (5)°	*T* = 293 K
γ = 101.865 (4)°	Block, colourless
*V* = 1609.93 (17) Å^3^	0.30 × 0.25 × 0.25 mm

### (II) Bis[N-(2-methoxyethyl)-N-methyldithiocarbamato-κ2S,S']diphenyltin(IV) Data collection

**Table:**

Agilent Technologies SuperNova Dual diffractometer with Atlas detector	8354 independent reflections
Radiation source: SuperNova (Mo) X-ray Source	6973 reflections with *I* > 2σ(*I*)
Mirror monochromator	*R*_int_ = 0.035
Detector resolution: 10.4041 pixels mm^-1^	θ_max_ = 30.4°, θ_min_ = 3.3°
ω scan	*h* = −10→10
Absorption correction: multi-scan (CrysAlis PRO; Agilent, 2015)	*k* = −19→19
*T*_min_ = 0.756, *T*_max_ = 1.000	*l* = −22→18
17063 measured reflections	

### (II) Bis[N-(2-methoxyethyl)-N-methyldithiocarbamato-κ2S,S']diphenyltin(IV) Refinement

**Table:**

Refinement on *F*^2^	0 restraints
Least-squares matrix: full	Hydrogen site location: inferred from neighbouring sites
*R*[*F*^2^ > 2σ(*F*^2^)] = 0.031	H-atom parameters constrained
*wR*(*F*^2^) = 0.078	*w* = 1/[σ^2^(*F*_o_^2^) + (0.0316*P*)^2^ + 0.0774*P*] where *P* = (*F*_o_^2^ + 2*F*_c_^2^)/3
*S* = 1.06	(Δ/σ)_max_ = 0.002
8354 reflections	Δρ_max_ = 0.66 e Å^−^^3^
338 parameters	Δρ_min_ = −0.56 e Å^−^^3^

### (II) Bis[N-(2-methoxyethyl)-N-methyldithiocarbamato-κ2S,S']diphenyltin(IV) Special details

**Table:**

Geometry. All esds (except the esd in the dihedral angle between two l.s. planes) are estimated using the full covariance matrix. The cell esds are taken into account individually in the estimation of esds in distances, angles and torsion angles; correlations between esds in cell parameters are only used when they are defined by crystal symmetry. An approximate (isotropic) treatment of cell esds is used for estimating esds involving l.s. planes.

### (II) Bis[N-(2-methoxyethyl)-N-methyldithiocarbamato-κ2S,S']diphenyltin(IV) Fractional atomic coordinates and isotropic or equivalent isotropic displacement parameters (Å^2^)

**Table:**

	*x*	*y*	*z*	*U*_iso_*/*U*_eq_	
Sn	0.53321 (2)	0.25616 (2)	0.25381 (2)	0.04036 (6)	
S1	0.34463 (8)	0.37273 (4)	0.23099 (4)	0.05095 (14)	
S2	0.75066 (8)	0.44734 (5)	0.23709 (5)	0.05550 (15)	
S3	0.22105 (8)	0.15139 (4)	0.26109 (4)	0.04817 (14)	
S4	0.53671 (8)	0.06041 (4)	0.27883 (4)	0.04702 (13)	
O1	0.3430 (3)	0.61622 (18)	0.10117 (16)	0.1049 (8)	
O2	0.6103 (3)	0.75288 (15)	0.38247 (15)	0.0899 (7)	
O3	−0.0963 (3)	−0.19679 (12)	0.15238 (12)	0.0705 (5)	
O4	0.1455 (3)	−0.09606 (15)	0.42343 (13)	0.0737 (5)	
N1	0.5046 (3)	0.55831 (13)	0.23494 (13)	0.0504 (4)	
N2	0.1846 (2)	−0.03099 (12)	0.27492 (12)	0.0426 (4)	
C1	0.5361 (3)	0.46932 (15)	0.23417 (14)	0.0437 (5)	
C2	0.3173 (4)	0.57560 (18)	0.22800 (18)	0.0601 (6)	
H2A	0.3202	0.6469	0.2604	0.072*	
H2B	0.2358	0.5348	0.2542	0.072*	
C3	0.2400 (4)	0.5493 (2)	0.1363 (2)	0.0750 (8)	
H3A	0.2446	0.4796	0.1022	0.090*	
H3B	0.1121	0.5544	0.1349	0.090*	
C4	0.2821 (6)	0.5939 (3)	0.0126 (3)	0.1359 (18)	
H4A	0.2869	0.5249	−0.0211	0.204*	
H4B	0.3609	0.6400	−0.0090	0.204*	
H4C	0.1575	0.6016	0.0082	0.204*	
C5	0.6601 (4)	0.64484 (18)	0.24329 (19)	0.0653 (7)	
H5A	0.6178	0.6911	0.2202	0.078*	
H5B	0.7560	0.6194	0.2093	0.078*	
C6	0.7398 (4)	0.70258 (18)	0.3363 (2)	0.0707 (8)	
H6A	0.7695	0.6555	0.3620	0.085*	
H6B	0.8526	0.7520	0.3387	0.085*	
C7	0.6699 (6)	0.8058 (3)	0.4717 (3)	0.1109 (13)	
H7A	0.7157	0.7615	0.4951	0.166*	
H7B	0.5680	0.8271	0.5018	0.166*	
H7C	0.7665	0.8647	0.4787	0.166*	
C8	0.3058 (3)	0.05076 (14)	0.27243 (13)	0.0379 (4)	
C9	−0.0177 (3)	−0.03932 (16)	0.27130 (16)	0.0485 (5)	
H9A	−0.0407	0.0288	0.2931	0.058*	
H9B	−0.0687	−0.0744	0.3092	0.058*	
C10	−0.1160 (3)	−0.09513 (17)	0.18215 (17)	0.0562 (6)	
H10A	−0.2459	−0.0940	0.1833	0.067*	
H10B	−0.0646	−0.0617	0.1432	0.067*	
C11	−0.1881 (5)	−0.2554 (2)	0.0696 (2)	0.0932 (10)	
H11A	−0.3185	−0.2603	0.0715	0.140*	
H11B	−0.1642	−0.3226	0.0516	0.140*	
H11C	−0.1445	−0.2235	0.0293	0.140*	
C12	0.2468 (3)	−0.11869 (16)	0.28315 (16)	0.0517 (6)	
H12A	0.3521	−0.1291	0.2503	0.062*	
H12B	0.1485	−0.1796	0.2581	0.062*	
C13	0.3001 (4)	−0.10467 (19)	0.37586 (18)	0.0602 (6)	
H13A	0.3463	−0.1626	0.3775	0.072*	
H13B	0.3977	−0.0436	0.4017	0.072*	
C14	0.1853 (5)	−0.0891 (3)	0.5099 (2)	0.0997 (11)	
H14A	0.2197	−0.1503	0.5095	0.150*	
H14B	0.0779	−0.0812	0.5404	0.150*	
H14C	0.2852	−0.0313	0.5383	0.150*	
C21	0.6777 (3)	0.32313 (15)	0.38043 (14)	0.0465 (5)	
C22	0.5984 (5)	0.3759 (3)	0.4491 (2)	0.0928 (11)	
H22	0.4791	0.3839	0.4411	0.111*	
C23	0.6951 (7)	0.4189 (3)	0.5326 (2)	0.1190 (14)	
H23	0.6389	0.4546	0.5795	0.143*	
C24	0.8694 (6)	0.4084 (3)	0.5450 (2)	0.0970 (11)	
H24	0.9330	0.4362	0.6001	0.116*	
C25	0.9485 (5)	0.3575 (3)	0.4769 (2)	0.0938 (10)	
H25	1.0692	0.3514	0.4846	0.113*	
C26	0.8533 (4)	0.3138 (2)	0.3950 (2)	0.0783 (8)	
H26	0.9102	0.2773	0.3489	0.094*	
C31	0.6273 (3)	0.18363 (15)	0.13244 (14)	0.0434 (5)	
C32	0.7940 (4)	0.1564 (2)	0.12717 (18)	0.0705 (7)	
H32	0.8682	0.1699	0.1777	0.085*	
C33	0.8533 (5)	0.1092 (2)	0.0478 (2)	0.0849 (9)	
H33	0.9680	0.0926	0.0452	0.102*	
C34	0.7435 (5)	0.0869 (2)	−0.0269 (2)	0.0854 (10)	
H34	0.7827	0.0541	−0.0802	0.102*	
C35	0.5772 (5)	0.1128 (3)	−0.02322 (19)	0.0885 (9)	
H35	0.5030	0.0979	−0.0741	0.106*	
C36	0.5176 (4)	0.1613 (2)	0.05655 (17)	0.0679 (7)	
H36	0.4037	0.1788	0.0588	0.081*	

### (II) Bis[N-(2-methoxyethyl)-N-methyldithiocarbamato-κ2S,S']diphenyltin(IV) Atomic displacement parameters (Å^2^)

**Table:**

	*U*^11^	*U*^22^	*U*^33^	*U*^12^	*U*^13^	*U*^23^
Sn	0.04240 (9)	0.04311 (9)	0.03293 (9)	0.01125 (6)	0.00010 (6)	0.00979 (6)
S1	0.0453 (3)	0.0431 (3)	0.0648 (4)	0.0074 (2)	−0.0022 (3)	0.0219 (3)
S2	0.0474 (3)	0.0555 (3)	0.0646 (4)	0.0082 (3)	0.0016 (3)	0.0251 (3)
S3	0.0418 (3)	0.0453 (3)	0.0620 (4)	0.0144 (2)	0.0033 (3)	0.0225 (3)
S4	0.0401 (3)	0.0506 (3)	0.0527 (3)	0.0140 (2)	0.0016 (2)	0.0195 (3)
O1	0.1032 (17)	0.1125 (17)	0.1005 (19)	−0.0200 (14)	−0.0375 (14)	0.0683 (15)
O2	0.0898 (15)	0.0732 (12)	0.0901 (16)	0.0321 (12)	−0.0235 (13)	0.0031 (11)
O3	0.0802 (13)	0.0559 (9)	0.0617 (12)	0.0067 (9)	−0.0153 (10)	0.0105 (8)
O4	0.0601 (11)	0.1001 (13)	0.0594 (12)	0.0032 (10)	−0.0044 (9)	0.0357 (11)
N1	0.0556 (11)	0.0430 (9)	0.0524 (12)	0.0066 (9)	−0.0052 (9)	0.0202 (8)
N2	0.0421 (9)	0.0402 (8)	0.0448 (11)	0.0111 (8)	0.0011 (8)	0.0136 (7)
C1	0.0486 (12)	0.0456 (11)	0.0344 (11)	0.0073 (10)	−0.0027 (9)	0.0136 (9)
C2	0.0646 (16)	0.0489 (12)	0.0689 (18)	0.0157 (12)	−0.0036 (13)	0.0227 (12)
C3	0.0669 (17)	0.0748 (17)	0.086 (2)	0.0054 (15)	−0.0199 (16)	0.0403 (16)
C4	0.133 (4)	0.164 (4)	0.107 (3)	−0.033 (3)	−0.053 (3)	0.087 (3)
C5	0.0717 (17)	0.0493 (13)	0.077 (2)	0.0013 (12)	−0.0020 (15)	0.0326 (13)
C6	0.0644 (16)	0.0480 (13)	0.090 (2)	−0.0012 (13)	−0.0163 (16)	0.0221 (13)
C7	0.108 (3)	0.094 (2)	0.100 (3)	0.030 (2)	−0.032 (2)	−0.003 (2)
C8	0.0403 (10)	0.0401 (10)	0.0314 (10)	0.0118 (9)	0.0005 (8)	0.0090 (8)
C9	0.0394 (11)	0.0484 (11)	0.0567 (15)	0.0088 (10)	0.0086 (10)	0.0182 (10)
C10	0.0425 (12)	0.0621 (14)	0.0610 (16)	0.0094 (11)	−0.0017 (11)	0.0205 (12)
C11	0.112 (3)	0.0767 (19)	0.065 (2)	−0.0041 (19)	−0.0185 (19)	0.0101 (15)
C12	0.0549 (13)	0.0387 (11)	0.0620 (16)	0.0134 (10)	0.0037 (12)	0.0172 (10)
C13	0.0572 (15)	0.0578 (13)	0.0698 (18)	0.0119 (12)	−0.0072 (13)	0.0296 (13)
C14	0.091 (2)	0.139 (3)	0.065 (2)	−0.006 (2)	−0.0112 (18)	0.051 (2)
C21	0.0552 (13)	0.0422 (10)	0.0364 (12)	0.0048 (10)	−0.0065 (10)	0.0113 (9)
C22	0.078 (2)	0.129 (3)	0.0483 (18)	0.027 (2)	−0.0008 (16)	0.0015 (18)
C23	0.134 (4)	0.152 (4)	0.0401 (19)	0.031 (3)	0.005 (2)	−0.002 (2)
C24	0.120 (3)	0.095 (2)	0.053 (2)	−0.011 (2)	−0.034 (2)	0.0211 (17)
C25	0.085 (2)	0.103 (2)	0.083 (3)	0.008 (2)	−0.038 (2)	0.032 (2)
C26	0.0730 (18)	0.093 (2)	0.0608 (19)	0.0302 (17)	−0.0145 (15)	0.0118 (15)
C31	0.0480 (12)	0.0468 (11)	0.0353 (11)	0.0125 (10)	0.0057 (9)	0.0135 (9)
C32	0.0602 (16)	0.102 (2)	0.0458 (16)	0.0309 (16)	0.0052 (13)	0.0150 (14)
C33	0.0707 (19)	0.113 (2)	0.070 (2)	0.0390 (19)	0.0278 (18)	0.0204 (18)
C34	0.099 (2)	0.102 (2)	0.0453 (18)	0.026 (2)	0.0291 (18)	0.0118 (16)
C35	0.097 (2)	0.124 (3)	0.0359 (16)	0.029 (2)	0.0013 (16)	0.0161 (16)
C36	0.0669 (16)	0.0914 (19)	0.0438 (15)	0.0282 (15)	−0.0009 (13)	0.0169 (13)

### (II) Bis[N-(2-methoxyethyl)-N-methyldithiocarbamato-κ2S,S']diphenyltin(IV) Geometric parameters (Å, º)

**Table:**

Sn—C31	2.124 (2)	C7—H7C	0.9600
Sn—C21	2.131 (2)	C9—C10	1.497 (3)
Sn—S1	2.5060 (6)	C9—H9A	0.9700
Sn—S3	2.5230 (6)	C9—H9B	0.9700
Sn—S4	2.9800 (6)	C10—H10A	0.9700
Sn—S2	2.9875 (6)	C10—H10B	0.9700
S1—C1	1.756 (2)	C11—H11A	0.9600
S2—C1	1.692 (2)	C11—H11B	0.9600
S3—C8	1.752 (2)	C11—H11C	0.9600
S4—C8	1.692 (2)	C12—C13	1.508 (4)
O1—C3	1.396 (3)	C12—H12A	0.9700
O1—C4	1.428 (4)	C12—H12B	0.9700
O2—C6	1.403 (3)	C13—H13A	0.9700
O2—C7	1.416 (4)	C13—H13B	0.9700
O3—C11	1.403 (3)	C14—H14A	0.9600
O3—C10	1.407 (3)	C14—H14B	0.9600
O4—C13	1.410 (3)	C14—H14C	0.9600
O4—C14	1.419 (3)	C21—C22	1.351 (4)
N1—C1	1.339 (3)	C21—C26	1.364 (4)
N1—C2	1.474 (3)	C22—C23	1.410 (5)
N1—C5	1.476 (3)	C22—H22	0.9300
N2—C8	1.337 (2)	C23—C24	1.355 (5)
N2—C12	1.472 (3)	C23—H23	0.9300
N2—C9	1.483 (3)	C24—C25	1.335 (5)
C2—C3	1.499 (4)	C24—H24	0.9300
C2—H2A	0.9700	C25—C26	1.383 (4)
C2—H2B	0.9700	C25—H25	0.9300
C3—H3A	0.9700	C26—H26	0.9300
C3—H3B	0.9700	C31—C32	1.370 (3)
C4—H4A	0.9600	C31—C36	1.383 (3)
C4—H4B	0.9600	C32—C33	1.381 (4)
C4—H4C	0.9600	C32—H32	0.9300
C5—C6	1.509 (4)	C33—C34	1.367 (5)
C5—H5A	0.9700	C33—H33	0.9300
C5—H5B	0.9700	C34—C35	1.359 (5)
C6—H6A	0.9700	C34—H34	0.9300
C6—H6B	0.9700	C35—C36	1.392 (4)
C7—H7A	0.9600	C35—H35	0.9300
C7—H7B	0.9600	C36—H36	0.9300
			
C31—Sn—C21	130.12 (9)	N2—C9—H9A	108.8
C31—Sn—S1	106.70 (6)	C10—C9—H9A	108.8
C21—Sn—S1	109.72 (6)	N2—C9—H9B	108.8
C31—Sn—S3	108.44 (6)	C10—C9—H9B	108.8
C21—Sn—S3	108.85 (6)	H9A—C9—H9B	107.7
S1—Sn—S3	82.873 (18)	O3—C10—C9	109.5 (2)
C31—Sn—S4	83.63 (5)	O3—C10—H10A	109.8
C21—Sn—S4	83.60 (6)	C9—C10—H10A	109.8
S1—Sn—S4	147.433 (18)	O3—C10—H10B	109.8
S3—Sn—S4	64.591 (16)	C9—C10—H10B	109.8
C31—Sn—S2	83.87 (5)	H10A—C10—H10B	108.2
C21—Sn—S2	81.92 (6)	O3—C11—H11A	109.5
S1—Sn—S2	64.922 (18)	O3—C11—H11B	109.5
S3—Sn—S2	147.742 (18)	H11A—C11—H11B	109.5
S4—Sn—S2	147.642 (17)	O3—C11—H11C	109.5
C1—S1—Sn	94.83 (7)	H11A—C11—H11C	109.5
C1—S2—Sn	80.40 (7)	H11B—C11—H11C	109.5
C8—S3—Sn	95.15 (7)	N2—C12—C13	112.95 (19)
C8—S4—Sn	81.36 (7)	N2—C12—H12A	109.0
C3—O1—C4	113.0 (3)	C13—C12—H12A	109.0
C6—O2—C7	113.3 (2)	N2—C12—H12B	109.0
C11—O3—C10	113.3 (2)	C13—C12—H12B	109.0
C13—O4—C14	112.2 (2)	H12A—C12—H12B	107.8
C1—N1—C2	122.75 (19)	O4—C13—C12	109.9 (2)
C1—N1—C5	120.5 (2)	O4—C13—H13A	109.7
C2—N1—C5	116.75 (18)	C12—C13—H13A	109.7
C8—N2—C12	121.08 (17)	O4—C13—H13B	109.7
C8—N2—C9	123.23 (17)	C12—C13—H13B	109.7
C12—N2—C9	115.69 (17)	H13A—C13—H13B	108.2
N1—C1—S2	122.83 (17)	O4—C14—H14A	109.5
N1—C1—S1	117.81 (17)	O4—C14—H14B	109.5
S2—C1—S1	119.36 (12)	H14A—C14—H14B	109.5
N1—C2—C3	113.2 (2)	O4—C14—H14C	109.5
N1—C2—H2A	108.9	H14A—C14—H14C	109.5
C3—C2—H2A	108.9	H14B—C14—H14C	109.5
N1—C2—H2B	108.9	C22—C21—C26	117.7 (3)
C3—C2—H2B	108.9	C22—C21—Sn	121.2 (2)
H2A—C2—H2B	107.8	C26—C21—Sn	121.12 (19)
O1—C3—C2	109.5 (2)	C21—C22—C23	120.5 (3)
O1—C3—H3A	109.8	C21—C22—H22	119.7
C2—C3—H3A	109.8	C23—C22—H22	119.7
O1—C3—H3B	109.8	C24—C23—C22	120.3 (3)
C2—C3—H3B	109.8	C24—C23—H23	119.8
H3A—C3—H3B	108.2	C22—C23—H23	119.8
O1—C4—H4A	109.5	C25—C24—C23	119.1 (3)
O1—C4—H4B	109.5	C25—C24—H24	120.5
H4A—C4—H4B	109.5	C23—C24—H24	120.5
O1—C4—H4C	109.5	C24—C25—C26	120.8 (3)
H4A—C4—H4C	109.5	C24—C25—H25	119.6
H4B—C4—H4C	109.5	C26—C25—H25	119.6
N1—C5—C6	112.0 (2)	C21—C26—C25	121.5 (3)
N1—C5—H5A	109.2	C21—C26—H26	119.2
C6—C5—H5A	109.2	C25—C26—H26	119.2
N1—C5—H5B	109.2	C32—C31—C36	118.6 (2)
C6—C5—H5B	109.2	C32—C31—Sn	121.56 (18)
H5A—C5—H5B	107.9	C36—C31—Sn	119.81 (17)
O2—C6—C5	109.2 (2)	C31—C32—C33	120.9 (3)
O2—C6—H6A	109.8	C31—C32—H32	119.5
C5—C6—H6A	109.8	C33—C32—H32	119.5
O2—C6—H6B	109.8	C34—C33—C32	120.0 (3)
C5—C6—H6B	109.8	C34—C33—H33	120.0
H6A—C6—H6B	108.3	C32—C33—H33	120.0
O2—C7—H7A	109.5	C35—C34—C33	120.1 (3)
O2—C7—H7B	109.5	C35—C34—H34	120.0
H7A—C7—H7B	109.5	C33—C34—H34	120.0
O2—C7—H7C	109.5	C34—C35—C36	120.1 (3)
H7A—C7—H7C	109.5	C34—C35—H35	119.9
H7B—C7—H7C	109.5	C36—C35—H35	119.9
N2—C8—S4	122.77 (15)	C31—C36—C35	120.2 (3)
N2—C8—S3	118.40 (15)	C31—C36—H36	119.9
S4—C8—S3	118.83 (11)	C35—C36—H36	119.9
N2—C9—C10	113.64 (18)		
			
C2—N1—C1—S2	176.85 (18)	C8—N2—C9—C10	95.7 (2)
C5—N1—C1—S2	−3.4 (3)	C12—N2—C9—C10	−85.3 (2)
C2—N1—C1—S1	−3.7 (3)	C11—O3—C10—C9	178.7 (2)
C5—N1—C1—S1	176.03 (18)	N2—C9—C10—O3	63.3 (2)
Sn—S2—C1—N1	173.13 (19)	C8—N2—C12—C13	84.5 (3)
Sn—S2—C1—S1	−6.27 (11)	C9—N2—C12—C13	−94.4 (2)
Sn—S1—C1—N1	−172.02 (16)	C14—O4—C13—C12	176.3 (2)
Sn—S1—C1—S2	7.40 (13)	N2—C12—C13—O4	62.5 (3)
C1—N1—C2—C3	−90.2 (3)	C26—C21—C22—C23	−0.4 (5)
C5—N1—C2—C3	90.0 (3)	Sn—C21—C22—C23	179.4 (3)
C4—O1—C3—C2	177.9 (3)	C21—C22—C23—C24	0.4 (6)
N1—C2—C3—O1	−66.0 (3)	C22—C23—C24—C25	0.6 (7)
C1—N1—C5—C6	−81.5 (3)	C23—C24—C25—C26	−1.6 (6)
C2—N1—C5—C6	98.3 (3)	C22—C21—C26—C25	−0.6 (5)
C7—O2—C6—C5	177.4 (2)	Sn—C21—C26—C25	179.6 (2)
N1—C5—C6—O2	−68.2 (3)	C24—C25—C26—C21	1.7 (5)
C12—N2—C8—S4	−0.3 (3)	C36—C31—C32—C33	1.1 (4)
C9—N2—C8—S4	178.62 (16)	Sn—C31—C32—C33	179.9 (2)
C12—N2—C8—S3	179.16 (16)	C31—C32—C33—C34	−1.5 (5)
C9—N2—C8—S3	−2.0 (3)	C32—C33—C34—C35	1.1 (5)
Sn—S4—C8—N2	177.09 (18)	C33—C34—C35—C36	−0.4 (5)
Sn—S4—C8—S3	−2.32 (11)	C32—C31—C36—C35	−0.4 (4)
Sn—S3—C8—N2	−176.72 (15)	Sn—C31—C36—C35	−179.3 (2)
Sn—S3—C8—S4	2.72 (13)	C34—C35—C36—C31	0.1 (5)

### (II) Bis[N-(2-methoxyethyl)-N-methyldithiocarbamato-κ2S,S']diphenyltin(IV) Hydrogen-bond geometry (Å, º)

**Table:**

*D*—H···*A*	*D*—H	H···*A*	*D*···*A*	*D*—H···*A*
C13—H13*A*···O2^i^	0.97	2.52	3.404 (4)	151

Symmetry code: (i) *x*, *y*−1, *z*.
